# The perspectives of nursing students regarding the incorporation of African traditional indigenous knowledge in the curriculum

**DOI:** 10.4102/phcfm.v12i1.2171

**Published:** 2020-04-16

**Authors:** Roinah N. Ngunyulu, Nombulelo Sepeng, Mabitja Moeta, Sanele Gambu, Fhumulani M. Mulaudzi, Mmampeko D. Peu

**Affiliations:** 1 Department of Nursing Science, Faculty of Health Sciences, University of Pretoria, Pretoria, South Africa

**Keywords:** curriculum, participatory transformative approach, African traditional Indigenous knowledge, practices, decolonisation

## Abstract

**Background:**

South Africa was caught off guard by the student unrest in 2015 and 2016. This unrest was named the #fees must fall campaign. During this campaign, students raised the issue of decolonisation of the curriculum, challenging the higher education fraternity and the academic community. This was based on the fact that the existing curriculum has inadequate content on African traditional indigenous knowledge (ATIK), and continues to use the Western approach to address the needs of a multicultural, multiracial and multi-ethnic societies. Institutions responded by initiating dialogues regarding decolonisation of the curriculum in senates, scholars and between different health professional bodies.

**Aim:**

This article aimed to explore and describe the perspectives of nursing students regarding incorporating ATIK into the curriculum.

**Methods:**

Using a participatory transformative approach, researchers and participants worked collaboratively to inform social change. Participants comprised nursing students. The academics, traditional health practitioners, indigenous knowledge holders and primary health care nurses formed the panellists. Data were collected through one communal dialogue workshop, which lasted for 8 hours, tea and lunch included. Data were analysed thematically.

**Results:**

Students’ perspectives emerged strongly as four themes, namely, politics of identity, displacement and distortion, curriculum content and institutional resistance. Students expressed that the current education system results in an identity crisis. The existing curriculum does not adequately convey an understanding of ATIK; it is displaced and distorted.

**Conclusion:**

Nursing science has great potential to incorporate the wealth of ATIK into its curriculum. In spite of a vibrant and rich cultural heritage, the ATIK specific to nursing sciences still needs to be incorporated into the existing curriculum in a responsive and relevant manner.

## Introduction

In South Africa, the *#Fees Must Fall* and #*Rhodes Must Fall* campaigns that took place in 2015 and 2016 raised multiple issues, including a call for decolonisation of the curriculum. This provoked a debate amongst academics and students. Even though these actions were not from nursing education *per se*, nursing students also formed part of the campaigns. In response, institutions, including those related to nursing education, were compelled to rethink the current curriculum. They were forced to consider how the curriculum addresses the needs of the people.^1^ Nursing education institutions also had to respond to this issue because they were responsible for the training of nurses who are the backbone of the health care system and are expected to provide compassionate care that will meet the health needs of patients from diverse cultural backgrounds. To meet the cultural health needs of all people living in South Africa, the curricula need to be reformed to restore the respective identities of previously marginalised and colonised groups who were affected in all facets of their lives.^2,3^ In South Africa, universities are still teaching curricula that are guided by colonial and apartheid worldviews. To decolonise and diversify this, curricula need to recognise African realism and include the lived experiences of most of the black people in South Africa.^4,5,6^

In the nursing sciences, nurses have always attempted to decolonise the curriculum by incorporating different cultures and accommodating cultural beliefs in teaching and learning as well as providing holistic nursing care. Holistic nursing care curricula are infused with theories such as cultural safety care, cultural diversity care and complementary alternative medicine.^7^ Ironically, the cultural theories and care that were incorporated in the nursing curriculum were developed by scholars in Europe and the United States. For example, Leininger’s cultural care theory and universality was developed in the United States and is used globally.^8,9,10,11,12^ These cultural theories are applied according to the unique health needs of people living in South Africa. Many of these theories that are commonly used in nursing are not always appropriate and relevant in an African cultural context.

As a point of departure, a culturally relevant curriculum should be consistent with the values, beliefs, worldviews and philosophies of the recipients of the intended knowledge.^13^ One of the dominant philosophies shared by almost all African indigenous people is *Ubuntu*, which forms the basis for caring within the African context. *Ubuntu* literally translates, ‘I am because you are.’ Although there is no one simple way of explaining the concept of *Ubuntu*, the principles of *Ubuntu* can be used to conceptualise the African view of health, illness and care in the health sciences.^13^ *Ubuntu* does not invalidate philosophies or theories from other worldviews but rather advocates for the recognition and unconditional, equal consideration of all worldviews informing societal values.^14^

In spite of calling for equal consideration of all worldviews, African traditional indigenous knowledge (ATIK) is often overlooked and not currently incorporated in the nursing curriculum. Added to that, several countries in Africa are practising indigenous knowledge systems in an attempt to achieve decolonisation within the health care system.^15^ In South Africa, the practice of alternative medicine is controlled by the Allied Health Professions Council of South Africa (AHPCSA). However, ATIK practices from Africa are not yet incorporated into the formal nursing curriculum.^16^ This is observed when patients are admitted to a hospital, with evidence of having used alternative medication or wearing traditional symbols of healing practices and traditional clothes; nurses do not have adequate knowledge and skills to understand and address the unique health needs of such patients. As a result, nurses are reticent to assist patients who are wearing traditional bands or who have traditional cuts. Ideally, nurses should have enough knowledge of traditional medicine to care for patients irrespective of the healing modality.^17^

In South Africa, communities are using traditional medicine and healing practices, such as traditional herbs, steam inhalation, purging, bone throwing, purification, animal sacrifices, healing sticks and other means, to appease the ancestors.^18^ Several strategies have been developed and introduced to recognise and incorporate ATIK practices into the South African health care system and into the curriculum. Amongst these strategies, the ‘African renaissance’ promoted the revival of local knowledge and brought research institutions, private cooperations, traditional healers and plant gatherers together to discuss issues. As part of the ‘African renaissance’, they also established a Commission for the promotion and protection of cultural, language and religious rights and to improve the quality of life by ensuring adequate health care provision.^18,19^ Another strategy is Primary Health Care reengineering (PHCr), which is aimed at the formal integration of complementary and alternative medicines within the country’s health care system.^20^ In South Africa, the *Traditional Health Practitioners (THP) Act* No. 22 of 2007 ensures that only trained and registered THPs under accredited institutions are at par with the Western health care practitioners.^21,22^

Nursing education institutions should prepare future nurses to deal with all health care modalities, to understand the principles of each modality and to advocate for their patients. In South Africa, this can only be done if relevant ATIK is incorporated into the nursing curriculum. After the #fees must fall campaign, nursing education institutions, amongst others, recognised the importance of including the perceptions of their students into the planning and development of curricula. In this article, we report on the perspectives of nursing students regarding the incorporation of ATIK into the nursing curriculum. This investigation uncovers whether the decolonisation of the curriculum is essential for nursing students.

## Research design and methods

The communal dialogue workshop followed a transformative participatory approach, which is qualitative in nature, to address nursing curriculum issues of inequality through open discussion, which initiated a communal dialogue between the nursing students and the expert panel members.^20^ Participatory transformative research is a continuous process and allows participants to be coresearchers. It contains an action agenda that aims at transforming the lives of individuals. In this study, it is meant to create awareness amongst nursing students regarding inequality and domination of Western knowledge over the ATIK in the nursing curriculum. The approach enabled students to unite their voices and account for their perceptions regarding the incorporation of ATIK in the nursing curriculum.^23^

### Research setting

This research was conducted at a university in the Tshwane District of South Africa. During the preparatory meeting for the workshop, this university volunteered to be the host. Nursing students from nine other South African universities volunteered to participate in the study.

### Population and participatory sampling methods

The population comprised 39 nursing students, 12 from the host institution and 27 from the other nine universities, who were registered during the research in 2018. Each university purposively selected their nursing students’ representatives, who were in their final year of study in 2018, and were interested in the topic and willing to participate in the study. The dialogue also included a panel of three academics (nurse educators) purposively selected from three local universities in the Gauteng Province. This was because they were experts in ATIK. Two THPs, who were experts in traditional healing practices; two indigenous knowledge holders, who were experts in indigenous knowledge; and one primary health care nurse, who also specialised in indigenous knowledge, were also included. The panel members were diverse in terms of gender, race, ethnicity, language, level of education and work experience. The purpose of the panel was to provide comprehensive information to the nursing students regarding the ATIK, to seek their different perspectives regarding its incorporation into the nursing curriculum.

### Data collection

Data were collected in a communal dialogue workshop. The communal dialogue was held in a circle to allow equal participation and full engagement with the subject matter. The researcher facilitated the communal dialogue, as she had expertise in conducting such dialogues. The workshop started with a presentation by one of the academics who gave comprehensive information about the current status of ATIK in the South African health care system. The purpose of the presentation was to stimulate the participants to think critically about ATIK and its relevance in the provision of quality health care to patients from diverse cultural backgrounds.

After the presentation, the facilitator focussed the communal dialogues around the main question, namely, ‘what are the perspectives of nursing students regarding the incorporation of the ATIK in the curriculum?’ This main question was directed at the participants. Nevertheless, in order for the participants to be able to answer the main question, individual expert academics, traditional health practitioners, indigenous knowledge holders and the primary health care nurses had to present their views on decolonisation by answering the following sub-questions:

- What does the concept decolonisation mean?
- What are the philosophies that should inform a decolonised curriculum?
- What is the envisaged decolonised curriculum?
- How will this curriculum be taught? By whom and where?

Each expert was given 15 min to present their views, followed by questions from the participants. The facilitator probed experts on their presentations, and this ensured that participants understood some of the concepts. The participants then reflected on the content that was presented and voiced their own perspectives through a communal dialogue with the panel of experts. Participants reflected on their preconceived ideas about what it means to be an African. Heated dialogues were conducted, and participants started to have an idea of the concepts of ‘decolonisation’ and ATIK. A dedicated scribe documented the perspectives on a chart visible to all in the venue. After the dialogue, the facilitator followed up with the participants to verify, clarify and make sense of certain connotations during the dialogues. The communal dialogue workshop lasted for 8 h, tea and lunch break included.

### Data analysis

Data were analysed concurrently with data collection. Participants worked with researchers to analyse and interpret data. The participants as co-researchers identified the following themes: politics of identity, displacement and distortion, curriculum content and institutional resistance.^24^ The participants analysed the data to identify themes.^25^ All participants concurred on the identified themes.

### Rigour in qualitative research

Measures to ensure trustworthiness were obtained through achieving relevant credibility, transferability, dependability, confirmability and authenticity. The credibility of the study was reached through prolonged and substantial engagement and member checking.^26,27^ Coresearchers spent about 8 h in a communal dialogue workshop, resulting in substantial engagement. During the communal dialogue workshop, participants had the opportunity to go through the identified themes, to verify if the data were captured accurately and sufficiently, ensuring member checking. Transferability was reached through keeping a dense description. Therefore, the coresearchers provided a dense description of the research context and methods.^28^ Dependability was also obtained by using a dense description of the study methods, particularly participative and transformative research design. The facilitator considered the perspectives, views, claims and voices of participants. The facilitator made it very clear that these aspects are visible and credited in the study.

### Ethical consideration

The permissions to conduct the study were also obtained from the deans and heads of the Departments of Nursing Sciences of all the universities that sent the nursing students and the panel members to the workshop. The written informed consent was obtained voluntarily from all the participants and the panel members, and they were treated equally and fairly with respect during the communal dialogue workshop. Confidentiality was ensured during data collection, analysis and interpretation.^23^

The Research Ethics Committee of the University of Pretoria, Ref number: 457/2017 approved the study.

## Results

Participants shared their perspectives regarding how the existing nursing curriculum has colonised them because it still follows the Western model of training. They indicated that the curriculum should be decolonised by incorporating components of ATIK. Four themes emerged: politics of identity, displacement and distortion, curriculum content and institutional resistance (see Table 1).

**TABLE 1:** The themes and subthemes that emerged from the group discussion.

Theme	Subtheme
Politics of identity	The poverty of the mind Cantering themselves in Africa Finding who they are
Displacement and distortion	Suspicion on ATIKs
Curriculum content	African philosophies to underpin curriculum design Incorporation of African history and literature on knowledge systems into curricula
Institutional resistance	Monolithic versus pluralism Coexistence and integration Status of current research ethical committee Science versus fiction

### Student perspectives regarding the inclusion of African traditional indigenous knowledge in the nursing curriculum

#### Politics of identity

Participants expressed that the current curriculum created an identity crisis. The identity crisis was expressed through the following subthemes: poverty of mind, centring themselves in Africa and finding who they are.

**The poverty of mind:** Participants indicated that they had a new understanding of poverty, namely, an intellectual ‘poverty of the mind.’ This poverty of mind was described in many ways, including the state of not being able to accept oneself the way one is. This was expressed by one of the participants: ‘We should teach our children that they are beautiful and that a bleaching agent is wrong.’

Participants expressed not feeling normal in their own skin colour, way of doing things and way of speaking. They felt compelled always to speak English using a Eurocentric accent. In addition, they perceived that normal behaviour should conform to Western standards. This was confirmed through the following statement:

‘Colonization of the mind starts from childhood; for instance, parents give teddy bears for their children to play with. Looking at the texture and colour of the skin of the bear, we are contaminating the minds of our kids.’

The poverty of the mind can also be seen in how Africans name their kid or child. In particular, historically English names were imposed upon Africans, and these Western practices have been assimilated without questioning. Over time, Africans have become comfortable with using European names.

One of the participants indicated that: ‘we gave our first-born child an African name, and as she was growing up she started asking why she was not given an English name.’

Participants reflected and summarised that the youth of today feel comfortable and a sense of belonging when they do things from a Western point of view. They expressed feeling ashamed to be associated with their own traditional norms, beliefs and practices.

One participant verbalised her perspective as follows: ‘… you will see it in everything we do such as dressing, health and eating in fact in everything … There is poverty of mind because of colonization.’

Most participants agreed and shared the same sentiments, and they attributed their colonised minds to the Western training approach.

**Cantering themselves in Africa:** Participants reflected that they had to centre themselves in Africa by learning and doing things in an African way. This perspective was supported by the following quotation: ‘we need proper assimilation of the African culture.’

Participants felt that there is a need to understand the politics of identity, which must be the core of the curriculum. Participants should understand where they are coming from, and the politics of identity should inform curriculum design to deepen their mindsets and help them discover how to centre themselves in the future.

Participants verbalised that the process of decolonisation could be commenced by finding a way to centre themselves in Africa so that they could view, believe, see and understand themselves as Africans. From the participants’ perspective, they argued that decolonisation could only take place if they knew and understood more about their own history and had insight into the damage caused by the apartheid regime. These perspectives are evident in the following quotes: ‘… we need proper assimilation and understanding of our history …’ we as South Africans we have been colonised, and the issue of apartheid came in which caused a lot of damages ….’

Another student said: ‘… the recommendation that I have is knowledge management, who manages the knowledge ….’

Participants felt there was a need to instil African values and beliefs to heal their colonised minds.

**Finding who they are:** Participants realised that they had been colonised; therefore, they had a need to rediscover their own history, so that they know where they are coming from, their current status and their future. They felt that the current curriculum was not empowering them with relevant ATIK. This perspective is evident in the following quote: ‘we have to discover ourselves. We also have to know that when colonization was taking place, distortion also took place.’

Participants felt that the curriculum is silent regarding the history of the ATIK. The existing curriculum promotes the history of Eurocentric nursing care modalities. Apart from colonisation, South Africans were also subjected to apartheid, which dubbed ATIK, in particular traditional healing, as witchcraft and promoted Christianity. Through the system of apartheid, Africans were made to be suspicious of each other and to believe that ATIK is inferior. This statement was confirmed when a participant raised the following question: ‘how do we accommodate traditional healers if they keep quiet and they don’t want to be seen in public to discuss issues that concern them?’

The secrecy around ATIK emanated from divisions promoted by both colonisation and apartheid. African scholars and students are now finding themselves seeking redress from the distortions of the past. They are in the process of finding who they are. One of the participants posed the following questions: ‘what does it really mean to be an African?’; ‘how do you decolonise health sciences?’

One participant who showed desperation and helplessness posed this question: ‘how do we discover ourselves?’ [*looking frustrated and emotional*].

#### Displacement and distortion of African traditional indigenous knowledge

The distortion and displacement of ATIK, as a result of colonisation, came out very strongly in this study. As indicated in previous themes, distortion and displacement happened because of colonialism, apartheid and imposition of Christian beliefs into nursing curricula by missionaries.

**Suspicion on African traditional indigenous knowledge:** Participants reiterated that although there is suspicion between ATIK holders and modern health care practitioners, both modes of practice should have space if nurses are to render holistic nursing care. The suspicions surrounding traditional health practices are fuelled by Christian beliefs, which have influenced nursing curricula and practise over time. Nurses embraced Christian views on nursing care and shunned other religious beliefs. One of the participants argued that: ‘even before Christianity, there were many religions, and both Christians and traditional healers believed in the fathers of Astrology who are the three wise men.’

This statement indicates that suspicion is worsened by the secrecy surrounding traditional health practices.

#### Curriculum content

Participants felt that existing curricula still perpetuated inequalities as nurses are still taught that modern medicine is the only legitimate source of health. Within this theme, two subthemes emanated, namely, African philosophies to underpin curriculum design and incorporation of African history and knowledge systems into curricula.

**African philosophies to underpin curriculum design:** The participants stressed that African philosophy must be used when designing a decolonised curriculum. The curriculum has to be relevant to health care provision. Some participants indicated that the things they were taught when growing up are distorted at a later stage in their training. This was stated as follows:

‘*Ubuntu* is one of those philosophies that can be used for the decolonisation of the curriculum. When you triangulate *Ubuntu* with all those others in your programme, you are likely to get good results.’

Apart from *Ubuntu,* feminism and humanism were also suggested as philosophies that could be used to incorporate ATIK into the curricula. However, there were vigorous debates as to whether feminism could be viewed as a philosophy to inform ATIK. One of the participants said this during the debate: ‘I have argued about humanism. Humanism is more Afrocentric than feminism. If you want to argue that you can check the origin of feminism.’

Seemingly, certain philosophies will only preserve the *status quo* or create imbalances, which may interfere with the process of decolonisation. In this instance, feminism is seen as a philosophy that encourages a division between men and women.

**Incorporation of African history and literature on knowledge systems into curricula:** Participants revealed that they lacked some basic knowledge of ATIK. The lack of knowledge was attributed to historical events associated with oppression and colonisation. There seems to be no trace of records on the contribution of Africans to modern medicine.

One participant said: ‘we need to deal with history of colonization and slavery. Our history of slavery and colonization should be the basis for transformation.*’*

Participants learnt that the lack of incorporation of African history, mainly related to nursing stalwarts and health practices, into the curriculum resulted in their lack of knowledge on ATIK.

#### Institutional resistance

Institutional resistance was one of the themes that came out strongly. Participants verbalised their frustration regarding how universities handle decolonisation of the curriculum and incorporation of ATIK into the curriculum. They felt that the pace of change is slow and in certain instances the process is not viewed as a priority. The subthemes that came up are monolithic versus pluralism, coexistence and integration; status of current research ethics committees, science versus fiction.

**Monolithic versus pluralism:** The participants verbalised that the curriculum is monolithic because it recognises modern medicine as the only mode of healing. They asserted that they had been taught nursing from a Western perspective only. They indicated that the panel discussion had opened their minds and helped them to start seeing things differently. The presence of the THPs and other advocates for integrating different modes of healing assisted the participants in changing their mindset. Participants realised that for nurses to provide holistic care, there is a need for nurses to be taught more than one mode of healing. They emphasised that they had been taught about cultural diversity care and complementary alternative medicine. However, traditional healing as a part of ATIK was not emphasised, and instead, they were taught about perceived harmful practices such as purging and the use of *isihlambezo* [herbal decoction used for induction of labour^27^] in midwifery.

One participant summarised this view when she said: ‘[*w*]e are here talking decoloniality tomorrow some health care science students will be told about cultural safety.’

The perception created in the current nursing curriculum is that ATIK is unsafe, unscientific and harmful. One participant supported the statement and said: ‘[*w*]e need to explore beyond only one source of knowledge if we really want to move forward.’

Participants questioned the monolithic way of looking at nursing by questioning the panellists, trying to understand the rationale behind the promotion of modern medicine accompanied by modern methods of nursing care as the only mode of care for patients. The following statement was uttered: ‘[*m*]odern way of thinking and practising care is dominant however if we want to move we have to consider other ways and methods of rendering care.’

**Coexistence and integration:** Students emphasised that conventional models of care would dominate ATIK, hindering decolonisation. A student used the following analogy: ‘[*i*]f you take 20 ml of orange juice and take five litres of pineapple juice. The pineapple juice will dominate orange juice.’

The analogy indicates that equal recognition and coexistence is necessary for both modern medicine and ATIK. Students felt that lack of proper integration is a barrier to rendering holistic care to patients.

**Status of current research ethical committees:** The ethical committees in health care faculties were identified as one of the impediments of progress in the generation of evidence-based ATIK. Master’s and PhD students who are interested in researching ATIK often have their protocols scrutinised, and their research questions and methods are often questioned and labelled as dubious.

This view was narrated by one of the panellists who said: ‘[*w*]hen you send your work to the ethics committee, they often say this methodology is not scientific.’

Findings illustrated that research ethics committees need to look beyond Western methodologies and consider other ways of knowledge creation.

**Science versus fiction:** The participants, who felt that it would be challenging to incorporate ATIK into the curriculum, as most of what is practised is not scientific, raised the issue of science versus fiction. They asserted that there are no books written about the trade and they felt that there is a lot of secrecy, which inhibits the exploration and documenting of traditional health practices.

## Discussion

In this study, participants confirmed that they have a political identity crisis, which they expressed as having poverty of the mind, finding who they are and an inability to centre themselves in Africa. This is based on the fact that the existing curriculum is based on the Western training approach, which does not include information on ATIK. Colonisation has also resulted in displacement and distrust of ATIK, which has resulted in a monolithic curriculum dominated by Western content. Participants also identified a need to create scientific content exploring the history and role of ATIK in modern medicine.

Decolonisation was one of the broader themes arising from the #fees must fall protests, impacting all areas of study. In South Africa, colonisation has been blamed for the poverty of the mind amongst participants, portrayed as ignorance of traditional values, beliefs and practices, and an inability to feel comfortable with their own skin colour. Through colonisation, Africans have distanced themselves from their own world.^29^ Participants expressed that poverty of the mind was evident in the choice of children’s names and Western food choices. Participants are keen to learn their own history, own cultural norms, values, beliefs and practices, which are currently not incorporated in the nursing curriculum to find themselves and centre themselves in Africa.

During the discussions, participants indicated a need to include African philosophy to underpin curriculum design. Africa and South Africa, in particular, have not been spared by the effects of these developments. Civil rights movements amongst students have demanded the restructuring of higher education institutions, with a critical focus on decolonising the curricula and culture within universities.^30^ As a result, dialogues have been initiated in many institutions in response to socio-political pressures associated with the demands for Africanised institutions of learning capable of meeting the diverse needs of the African population.

During discussions, participants expressed that traditional medicine is mired in secrecy. Traditional healers, in particular, felt that their knowledge was their intellectual property, and subsequent livelihood. This poses challenges to the sharing and documenting of ATIK. The recipes of remedies and herbs used by traditional healers are not divulged to those who are not practising traditional healing.^30,31^ In addition, the indigenous knowledge amongst THPs is often passed on verbally to ensure secrecy of the doctrines.^31^ This lack of sharing and documentation may be partly to blame for participants not having access to ATIK.

Chitindingu, George and Gow indicate that health sciences students still lag behind when it comes to ATIK and traditional medicine.^32^ In this study, participants indicated that they were not aware of many aspects of traditional healing. In instances where participants received training on traditional healing, the approaches are fundamentally theoretical and did not expose participants to real problems.^33^ Participants also expressed a need to learn the history of ATIK and related literature. Apart from a lack of sharing, historical events have led to the domination and, in some cases, total eradication of indigenous health systems across many countries. The development of Western medicine has been driven by Western countries dominating vulnerable societies.^16^ European settlers sought to intentionally destabilise indigenous societies by banning traditional healing, forcing indigenous groups to rely on Western medicine and thereby displacing traditional healing.^34^ In Africa, colonisation has resulted in widespread *epistemicide*, which refers to the total eradication and possible falsification of any source of knowledge amongst the colonised indigenous people.^35^ Undoing the effects of colonialism requires an honest confrontation of the damages, as well as holding institutions accountable.^5^ This should be the basis for the decolonisation of curricula and transformation within institutions of learning.

Participants identified institutional resistance as an impediment to curriculum reform. An opposition to change is entrenched in university structures where racism, sexism, Eurocentric epistemologies and pedagogical practices are often the order of the day.^36^ Current structures such as university councils, senate, professional bodies and curriculum committees are responsible for curriculum design and review. In instances where decolonisation of the curriculum remains a concern, institutions must take the lead in decolonisation. Institutions should be held accountable by higher authorities regarding the mechanisms put in place to transform the curriculum.^5^

The higher education systems are often marred by power struggles, privileges and patronage as well as dominated by scholars who have been socialised through the same system. They are used to hierarchical structures, and it is difficult for them to understand the inclusion of the participants in the discussion of curriculum issues. Jansen reported that the students who attend curriculum committees often do not attend meetings as expected.^37^ Their absence and perceived lack of commitment may affect progress and also stifle the debates that are expected in those forums.^37^ However, Joseph argues that students are often faced with issues of power relations between themselves, lecturers and management structures, which can contribute to their absenteeism and inactivity in such meetings.^38^

Participants raised concerns regarding a monolithic nursing curriculum, which promotes unilateralism and the supremacy of Eurocentric views. Pluralism remains a challenge in the health care system that overlooks other alternative methods of care. South Africa is still predominantly using a single method of health care delivery, a view supported by Jansen who argues that the ‘domination of western knowledge has marginalised, even museumified, alternative knowledge.’^37^ Gail and Mbamalu identified a proposal of restructuring primary health care as a perfect opportunity for complementary and alternative medicine to be formally integrated into the country’s health care system. However, they were not clear if restructuring would include ATIK.^39^ In South Africa, decolonialist groups have argued that there has been no curriculum transformation since 1994, but Jansen pointed out that there was remarkable curriculum reform towards the promotion of ATIK led by prominent African scholars. Participants in this study have seen little of such reform.^37^

Participants expressed a need for coexistence and integration of Western medicine and ATIK. In a country such as South Africa, where multiple and diverse cultures exist, recognition and mutual respect for the practised cultures is imperative. The *South African Constitution Act* assures all citizens that their cultural practices will be protected and not discriminated against.^40^ However, there has been little progress in effecting this aspect of the Constitution, as evidenced by the persistent and frequent undermining of ATIK in all fronts of government. Although the *THP Act* No. 22 of 2007 was promulgated in an effort to formalise and regulate the practice of THPs, the government has not demonstrated its commitment to include ATIK in higher education curricula.^23,41^ African traditional indigenous knowledge is still alienated and subjected to rigorous interrogations, especially within the health care system, indicating the negative attitude harboured towards traditional healing.^42^

It is clear that stereotypes and labelling of ATIK as witchcraft is negatively affecting nurses’ ability to render holistic, non-discriminatory health services as stipulated in the patient’s right charter.^43^ Consequently, the patient’s right to access culturally sensitive health care services is impeded. Seamless integration will require mutual respect, advocacy at all levels of government and a commitment for working together by the health care workers and THPs. The government and health institutions should intensify efforts and speedily create platforms for dialogue on how to give ATIK an equal piece of the cake and not be dominated or seen as second best.

Many countries, globally and in Africa, have demonstrated that coexistence is possible. For example, in China, the Chinese traditional medicine is at par with modern medicine. In fact, the Chinese government facilitated the integration and total incorporation by criminalising unjust criticism and the deliberate undermining of traditional Chinese medicine.^44^ This demonstrates the critical role of governments in giving ATIK space and resources to excel. South Africa could learn a lesson or two from such cases because most of the population still uses traditional medicine as a source of health support.

## Limitations

The study only used nursing students who volunteered because of an interest in the topic, so the results of this study cannot be transferred to other students who have registered for other health care sciences programmes.

## Conclusion

Participants indicated a need for the decolonisation of health care sciences curriculum to provide holistic care amongst patients who consult within the health care system. Holistic care for a diverse South African population can only be achieved when the two systems, namely, Western medicine and ATIK, are integrated and equally recognised. Decolonisation of the health care curriculum will help nursing students to know about the history of South African health care and how conditions and diseases are managed using ATIK. This historical knowledge will help students to build their identity and accept themselves as Africans.
